# Community reporting of ambient air polychlorinated biphenyl concentrations near a Superfund site

**DOI:** 10.1007/s11356-017-0286-6

**Published:** 2017-10-27

**Authors:** Kathryn S. Tomsho, Komal Basra, Staci M. Rubin, Claire B. Miller, Richard Juang, Sylvia Broude, Andres Martinez, Keri C. Hornbuckle, Wendy Heiger-Bernays, Madeleine K. Scammell

**Affiliations:** 1 Department of Environmental Health, Boston University School of Public Health, 715 Albany St., Talbot 4W, Boston, MA 02118 USA; 2 Alternatives for Community and Environment, 2201 Washington St. 3302, Roxbury, MA 02119 USA; 3 Toxics Action Center, 294 Washington St. #500, Boston, MA 02108 USA; 4 Department of Civil and Environmental Engineering, IIHR-Hydroscience and Engineering, 4105 Seamans Center for the Engineering Arts and Sciences, The University of Iowa, Iowa City, IA 52242 USA

**Keywords:** Report-back, Community engagement, Polychlorinated biphenyls, Ambient air, Research translation, Environmental data communication

## Abstract

In this manuscript, we describe the process of establishing partnerships for community-based environmental exposure research, the tools and methods implemented for data report-back to community members, and the results of evaluations of these efforts. Data discovery and report-back materials developed by Statistics for Action (SFA) were employed as the framework to communicate the environmental data to community members and workshops. These data communication and research translation efforts are described in detail and evaluated for effectiveness based on feedback provided from community members who attended the workshops. Overall, the methods were mostly effective for the intended data communication.

## Introduction

New Bedford Harbor (NBH) in Massachusetts stretches across 18,000 acres in the Acushnet River estuary and Buzzards Bay. In 1983, NBH was listed as a national Superfund site due to the harbor-wide contamination by polychlorinated biphenyls (PCBs) and heavy metals (District, 2015). PCBs were used in industrial processes until the 1970s, when they were banned by the United States Environmental Protection Agency (US EPA). In NBH, the primary source of the PCBs was the Aerovox facility, formerly located in the Upper Harbor (Superfund, 2014). The majority of PCBs exist on the harbor floor, bound to sediment. In 1998, the US EPA first announced its plans to mitigate the PCB-contaminated sediment. After hot spots were removed and taken off-site, areas of concern would be dredged and then placed within confined disposal facilities, which are engineered structures that isolate dredged material from surrounding water for containment, with the goal of reducing concentrations to meet guidelines that are intended to improve local environmental health (District, 2015).

The primary exposure to PCBs considered to pose a risk to human health is from consumption of locally caught seafood (U.S. Environmental Protection Agency, 2015). However, residents in the surrounding communities of New Bedford, Fairhaven, Dartmouth, and Acushnet are concerned about exposure to PCBs via ambient air. Of particular concern to residents affiliated with a local grassroots community group, Hands Across the River Coalition (HARC), is the impact of the remedial dredging on air quality. HARC sought scientific and legal assistance from Alternatives for Community and Environment, Inc. (ACE) who engaged environmental health professionals at Boston University’s Superfund Research Program (BUSRP) (Boston University Superfund Research Program, 2016b). To address HARC’s concern, BUSRP and ACE partnered with Toxics Action Center (Toxics Action) and the University of Iowa Superfund Research Program (Iowa SRP) to seek funding for further investigation of airborne PCB exposure. *PCBs in Ambient Air: Responding to Community Concerns around New Bedford Harbor* is a study funded by the National Institute of Environmental Health Sciences (NIEHS/NIH) Superfund Research Program, designed to evaluate the contribution of PCBs from the NBH waters to the surrounding community in response to concerns raised by the local community regarding the PCBs in the air during dredging of the sediments in the harbor. A central goal of this project was to empower HARC members so they understood and took ownership of the data collection process. Toxics Action, ACE, and BUSRP worked together to develop materials and workshops that would assist air monitor hosts with the logistics of collecting air samples, answer related questions, and understand how the data they helped to collect compares to other environmental data collected around NBH.

Materials from Statistics for Action (SFA) were tailored to incorporate these data and used at two NBH workshops with air monitor hosts in February 2016. SFA is the product of a grant awarded to Toxics Action and Technical Education Research Centers (Statistics for Action, 2014a). To create SFA, Toxics Action and Technical Education Research Centers asked the Community Engagement and Research Translation leaders of BUSRP to serve as advisors and consultants, supported by a National Science Foundation grant. The goal was to provide community members and environmental organizers with frameworks and resources to assist communities impacted by environmental degradation to analyze, understand, and effectively communicate potential local health risks based on scientific data. SFA’s tools include guidebooks, online videos, and an informational website accessible for community members, environmental professionals, and organizers (Statistics for Action, 2014b).

We describe the history and context of the partnership for our community-based environmental exposure research, the tools and methods implemented for data report-back to community members, and our evaluation of these efforts. With 74 active PCB-contaminated Superfund sites across the USA ( US EPA 2016) and PCBs in school air (Herrick et al., 2004; 2016; Marek et al., 2017; Office of Senator Edward J. Markey, 2016), the process used in NBH can inform future efforts to engage communities affected by airborne PCBs in their communities.

## Methods

### Establishing the partnerships

The collaborative work of Boston University Superfund Research Program (BUSRP), Iowa Superfund Research Program (Iowa SRP), Toxics Action Center (Toxics Action), Alternatives for Community and Environment (ACE), and Hands Across the River Coalition (HARC) was a result of several long-standing relationships and some new connections. The Superfund Research Program has existed through funding made possible by NIEHS since 1986. This program was established under the Superfund Amendments and Reauthorization Act, and funds higher education institutions across the USA to perform research related to human and environmental health concerns pertinent to contaminants commonly found at Superfund sites. BUSRP has existed since 1995. The BUSRP includes Community Engagement Core (CEC), which facilitates bi-directional relationships between affected communities and SRP investigators, and Research Translation Core (RTC) that translates SRP research to key government stakeholders. BUSRP investigators, CEC, and RTC leaders and partner organizations have studied NBH contaminants and worked with area residents on a variety of topics since the BUSRP was established.

Toxics Action Center has been a partner of the BUSRP since 2000. Toxics Action is a New England-based public health and environmental non-profit that works side by side to build the organizing capacity of local community groups. Founded in 1987, their staff has expertise working closely with community groups on environmental health-based concerns and specializes in community organizing, strategizing, and leadership development.

Alternatives for Community and Environment (ACE), established in 1993, has been an SRP partner since 2006 and is a Massachusetts-based organization that works specifically with low-income communities and communities of color to address injustice and achieve environmental justice.

Hands Across the River Coalition (HARC) is a New Bedford-based grassroots environmental advocacy group. Founded in 1982, HARC has advocated for a harbor clean up that is protective of local community health and has strived to educate the local residents of events and issues related to the cleanup. At various times over the last 20 years, HARC has worked informally with both Toxics Action and ACE, as well as leaders of the BUSRP.

**Fig. 1:**
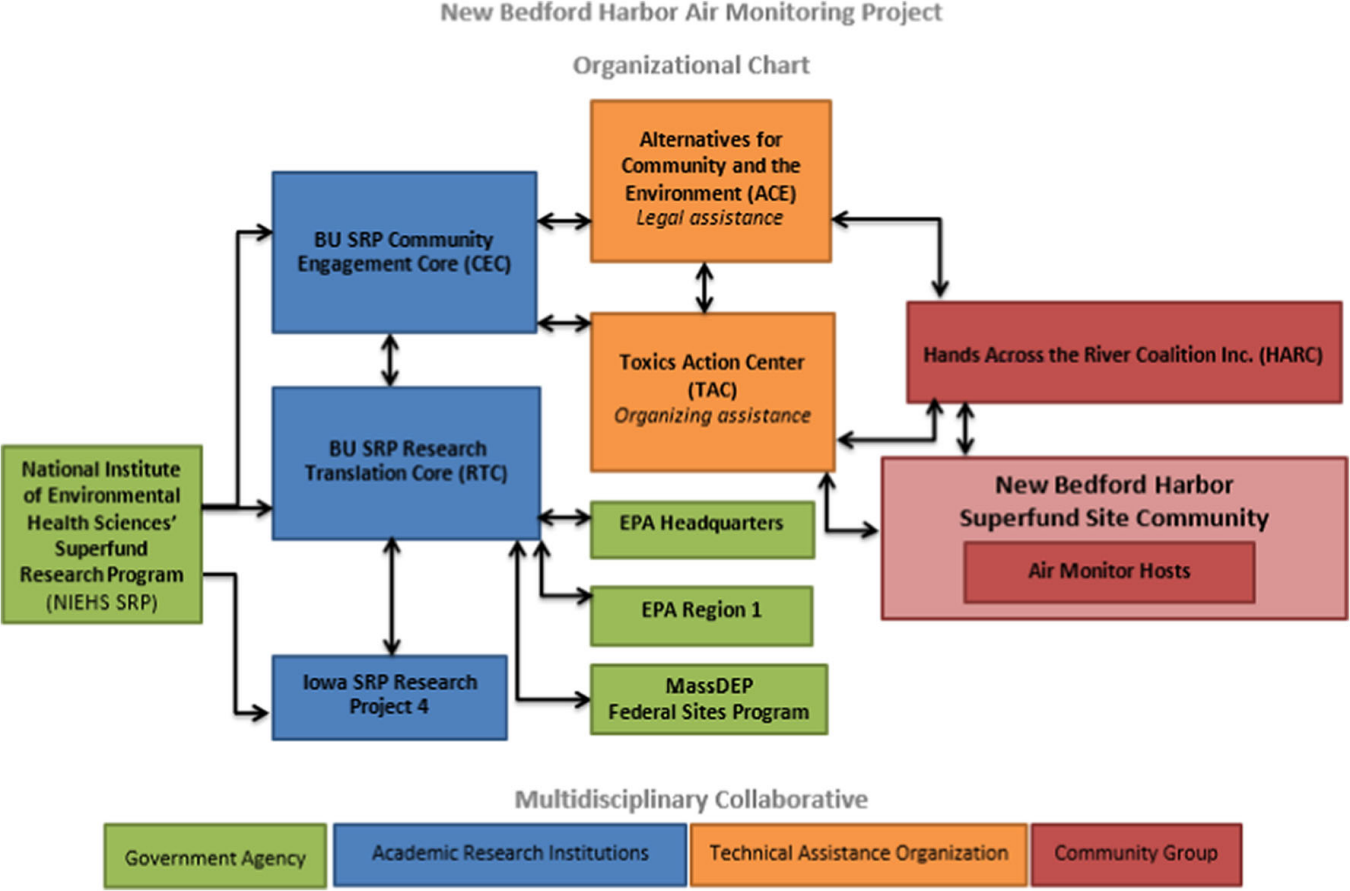
Organizational roles and connections

In this instance, ACE worked with HARC to develop research questions related to NBH and shared these questions with BUSRP. BUSRP engaged researchers at Iowa SRP, which has over a decade of experience studying lower molecular weight PCBs and their sources (Robertson, 2012). One Iowa SRP project focuses specifically on identifying and measuring atmospheric sources of individual PCB congeners using passive air-monitoring apparatuses. Their facilities include an on-site laboratory for in-house gas chromatography with mass spectrometry (GC-MS/MS) analysis for individual PCB congeners measured in the field (Ampleman et al., 2015; Martinez et al., 2017). Each of the involved organizations and their roles are displayed in Fig. 1. BUSRP’s role was to design the study, manage the air monitors, and analyze the results. Toxic Action Center’s role was to facilitate conversations with air monitor hosts and communicate the study results. ACE’s role was to provide legal assistance, as necessary, and review written materials. Iowa SRP’s role was to generate the raw data from the air monitors. HARC’s role was to recruit air monitor hosts and serve as the convener of residents and stakeholders interested in NBH environmental health concerns. These five organizations served as project leaders.

### Site identification and PCB data collection

**Fig. 2:**
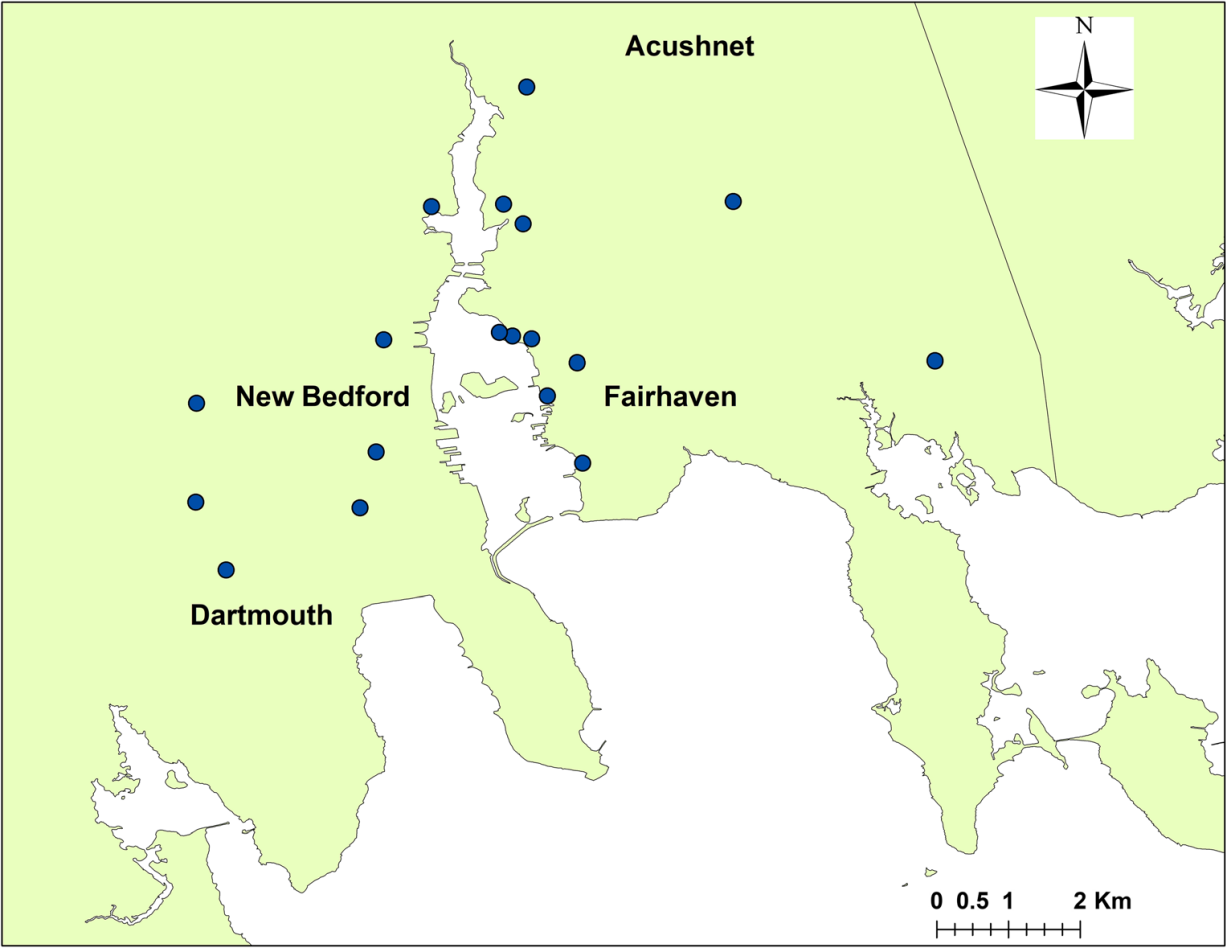
Ambient air monitoring locations around NBH

In spring of 2015, all partners, including Iowa SRP, traveled to Fairhaven for a meeting with concerned residents hosted by HARC. Iowa SRP demonstrated how the passive air monitors function and explained that to obtain a representative understanding of the ambient air PCB concentrations across the harbor, the monitors needed to be placed in transects across the NBH region, at varying distances inland from the water. Using a large area map of the harbor, Toxics Action community organizers then worked with HARC members to identify potential locations. Several properties belonged to HARC members who hoped to learn about PCB exposure. Once these locations were identified on the map, areas in which additional monitors would be of interest were discussed, and HARC members called friends and neighbors to recruit other monitor hosts. Some, but not all, monitoring locations were confirmed at this meeting. Others were identified over the following weeks. Ultimately, 18 locations, at homes and businesses where there was an English language speaker, were confirmed throughout Dartmouth, Acushnet, New Bedford, and Fairhaven, as shown in Fig. 2.

**Fig. 3:**
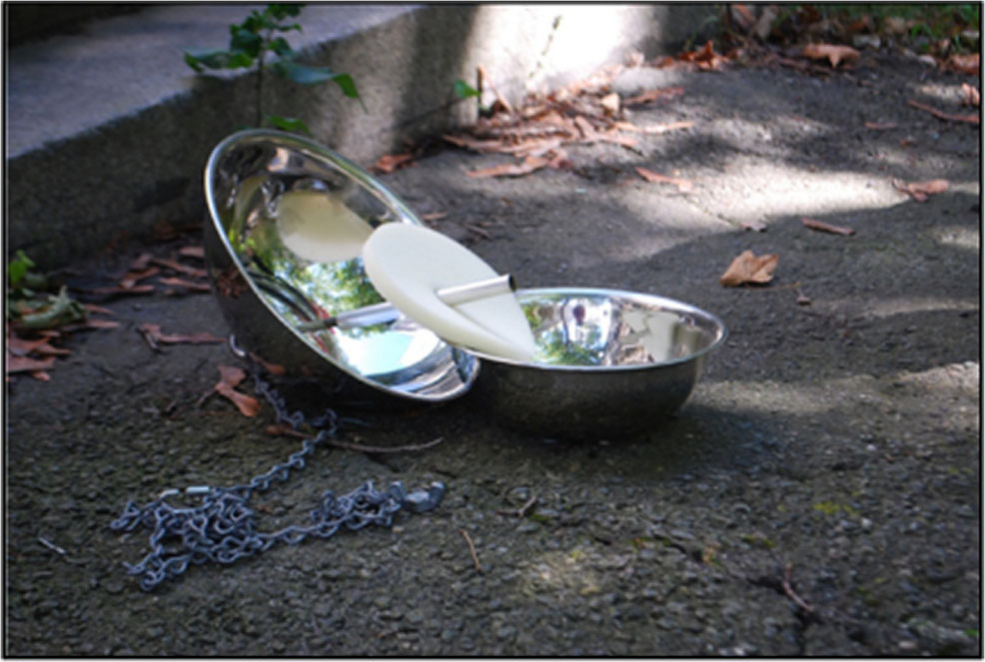
Passive monitoring apparatus for PCBs in ambient air

Passive air monitor shells were installed over the course of 2 days in late June 2015. A metal chain attached to the monitors was wrapped around tree branches, flag poles, chain-link fences, or Shepard’s poles. The monitor shells were constructed of two metal bowls with a rod through the center, as shown in Fig. 3. A Monarch Instrument Track-It Data Logger was taped to the inside of the shell to collect temperature and humidity data. The sensor on the Track-It Data Logger faced towards the center of the monitor to ensure it did not touch the metal sides. A polyurethane foam (PUF) was pierced by the rod and left to hang in the center of the shell. PUFs were provided by Iowa SRP, wrapped in foil, and mailed overnight in individual Ziploc bags to BUSRP. The PUFs were received at BUSRP the day before installation in the field and were stored in a − 80 °C freezer overnight. PUFs collected in the field after 6 weeks were again wrapped in foil, individually stored in Ziploc bags, and overnight shipped back to Iowa SRP for analysis. Iowa SRP’s methods for analysis have been described in other publications (Ampleman et al., 2015; Herkert et al., 2016; Martinez et al., 2017) (US EPA 2010).

For this project, four rounds of PUFs were installed, each for 6 weeks durations. The first three rounds were consecutive—beginning in July of 2015 and ending in November of 2015 to capture the warm and colder season months prior to active dredging of contaminated sediment in the harbor. The fourth round of collection occurred from July to August 2016 exactly a year later in a warm season but while there was active dredging of contaminated sediment. For each round, all PUFs were installed on the same day. As each location’s PUF was collected, a new PUF was installed for the subsequent round.

### Data sharing with the community

Within a month following the second round of data collection, Iowa SRP provided results to BUSRP from the first two rounds of data collection. Upon receipt of the first two rounds of data, members of Toxics Action, ACE, and BUSRP met to review the raw data and to discuss approaches to presenting the data to the monitor hosts. Toxics Action staff led the first workshop using SFA’s First Look at Technical Documents: Environmental Tests Results materials to review the first two rounds of data (Statistics for Action, 2014a). The second workshop followed 2 weeks later, allowing time for BUSRP, Toxics Action, and ACE to develop educational materials, incorporate the recently received third round of data, and include responses to questions raised at the first workshop. Iowa SRP provided results from the fourth round of data in late summer of 2016. BUSRP, Toxics Action, and ACE communicated the results via mailings containing information requested by participants in the first two workshops.

SFA suggests the use of First Look at Technical Documents: Environmental Test Results materials when a community receives raw data from environmental testing and needs a starting point from which to approach the data (Statistics for Action, 2014a). SFA also suggests that through this process, the community organizers leading the workshop may develop a better understanding of the needs of the community for additional data interpretation and ownership, and better identify additional SFA materials that can be used moving forward (Statistics for Action, 2014a).

Using an activity in which participants examine the raw data as a starting point and SFA materials, BUSRP and Toxics Action presented the data to the air monitor hosts as printed spreadsheets and provided them with sticky notes. Participants looked through the data, identified questions or comments, and wrote them on a sticky note. The facilitators (from BUSRP and Toxics Action) then moderated a discussion with the group to categorize their questions into themes and to place the sticky notes with those questions into appropriate clusters by theme on the walls. During the debriefing, air monitor hosts discussed their reactions to the dataset and identified aspects that they thought were of interest or importance. Finally, facilitators helped identify next steps, spanning from defining terms, to identifying future research or experts that could assist with further data analysis in a second workshop (Statistics for Action, 2014a).

### Workshop evaluations

Evaluations were distributed after both workshops to all attendees. The one-page questionnaire asked the following questions: After this session, do you feel more able to understand the role numeric data plays in regulations and guidelines on ambient PCBs? If yes, what about the workshop made you feel more able? What is the most interesting or valuable thing you are taking from the session? How will it be useful to you? It also asked participants to rate their level of agreement or disagreement to the following statements on a Likert scale (Sullivan and Artino, 2013): I gained experience understanding congeners, homologs, and Aroclors. I gained skills to interpret regulations and guidelines on ambient PCBs. I became more confident comparing the data from this project and the US EPA data on ambient PCBs. Response options included strongly agree, agree, neutral (neither agree or disagree), disagree, and strongly disagree.

Facilitators collected evaluations from participants and compiled results in an excel document for later review by BUSRP members.

### Data report back mailings

All participants were mailed two rounds of information that included their location’s monitoring results, as well as a fact sheet on PCBs (Boston University Superfund Research Program, 2015), and a visual explaining the passive monitoring used in this project compared with the active 24-h monitors used by the US EPA (Boston University Superfund Research Program, 2016a).

### Host interviews

Five months after the second data report-back mailings were sent, monitor hosts were contacted via email and/or telephone call to participate in a voluntary interview to evaluate the communication efforts. Hosts who volunteered were interviewed via telephone. They were asked to describe their memory of the packets and to provide ideas about improving the materials. Project leaders also asked whether hosts attended the data workshops and what lead them to attend or not attend.

## Results

### First Look at Technical Documents workshop

Only air monitor hosts were invited to attend workshops. There were ten attendees at the first data workshop. Although the meetings were intended to be limited to monitor hosts, one host also brought a concerned neighbor to the meeting. The workshops were conducted in English. All workshop participants speak English. Of the monitor hosts, six were members of Hands Across the River Coalition (HARC) before the start of this project. Before the data sheets were shown to attendees, several monitor hosts voiced their concern as to what they would learn about their own ambient air concentrations. One host said, “As hosts, we are very close to the situation. We are very anxious to find out what our data are”.

To implement the Statistics for Action (SFA) First Look at Technical Documents workshop, Boston University Superfund Research Program (BUSRP) members printed the dataset for each of the 18 monitoring sites’ first two rounds of ambient air PCB concentrations. The data set included the total PCB concentrations (∑PCBs ng/m^3^) and individual congener concentrations for each monitoring location. Once printed, the dataset spanned across a large printed spreadsheet, as shown in Fig. 5. At the meeting, Toxics Action Center (Toxics Action) organizers described the data activity and provided each participant with sticky notes and markers. For approximately 45 min, participants reviewed the data. Some worked alone, but most worked in pairs to parse through the information together. Some participants had expected that the data would be interpreted and presented back to them by the BUSRP team. When given the multi-page raw data spreadsheet, one monitor host became flustered, saying, “This looks like we’re looking at an EPA chart. These are statistics-I don’t know statistics!” This feeling was shared by many in the room. After several minutes of coaxing, the participant was convinced to take part in the initial data discovery activity with the promise that everyone would work together to understand the data.

**Fig. 4:**
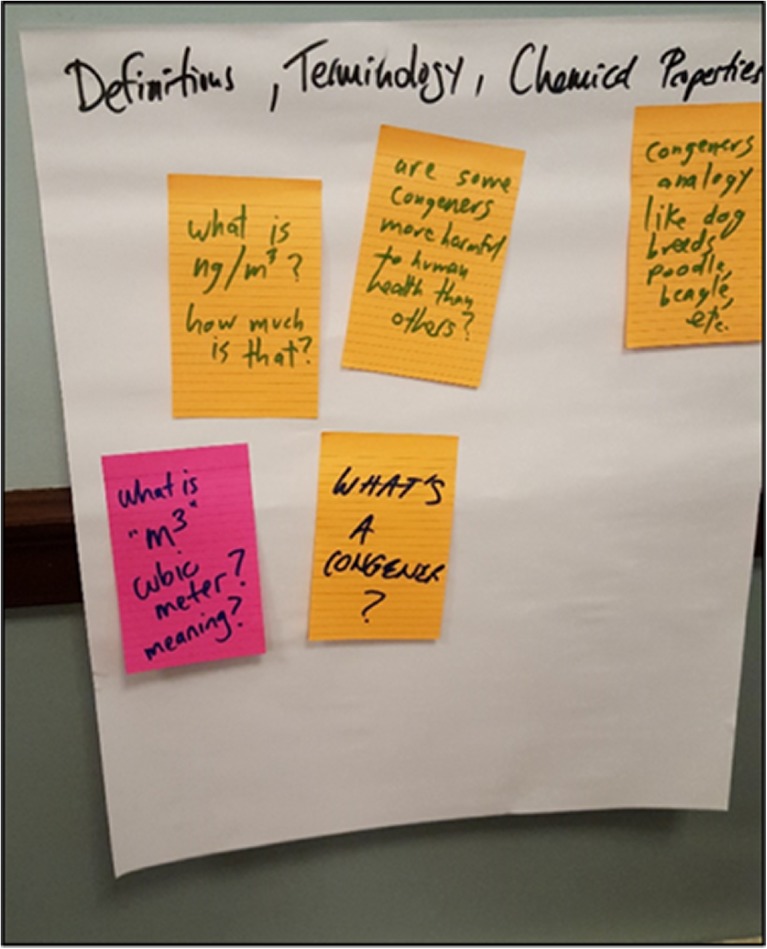
Workshop facilitators organized monitor hosts’ questions by theme

As participants worked together, they identified 50 questions, reflecting a wide range of familiarity with PCBs and environmental datasets. As the hosts absorbed the dataset and identified their questions, Toxics Action organizers and BUSRP members circulated to answer immediate questions and collect their sticky notes with questions. Toxics Action organizers then posted large tablet papers with question categories around the meeting room to help organize the questions, as shown in Fig. 4. Question categories included the following: “The Testing Process;1” “Definitions, Terminology, and Chemical Properties;” “Results as Presented in Report;” “Human Health Risk;” “What Action Should Happen Now;” and “Other.” Initial questions and comments centered on the basic characteristics of the dataset: “What’s bad and what’s good?”; “Why are some higher and some lower?”; “What is ng/m^3^?”; “Is that like an eighth of a teaspoon?”

**Fig. 5:**
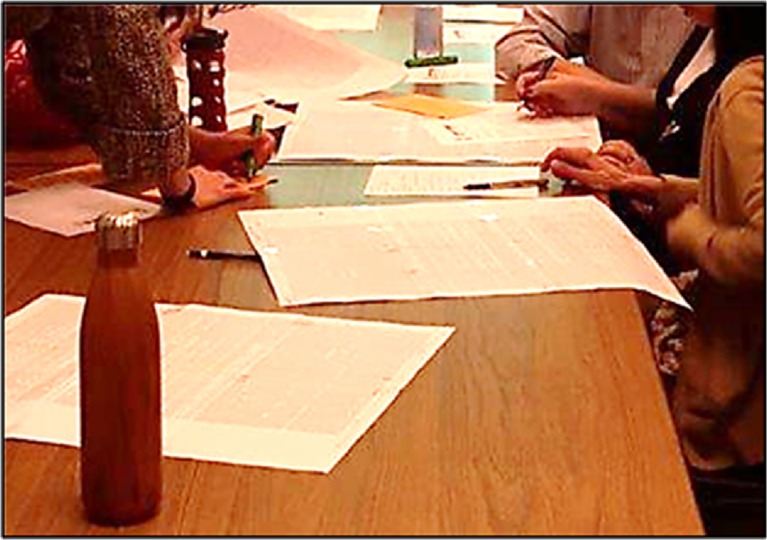
Workshop participants review raw ambient air PCB concentration data

**Fig. 6:**
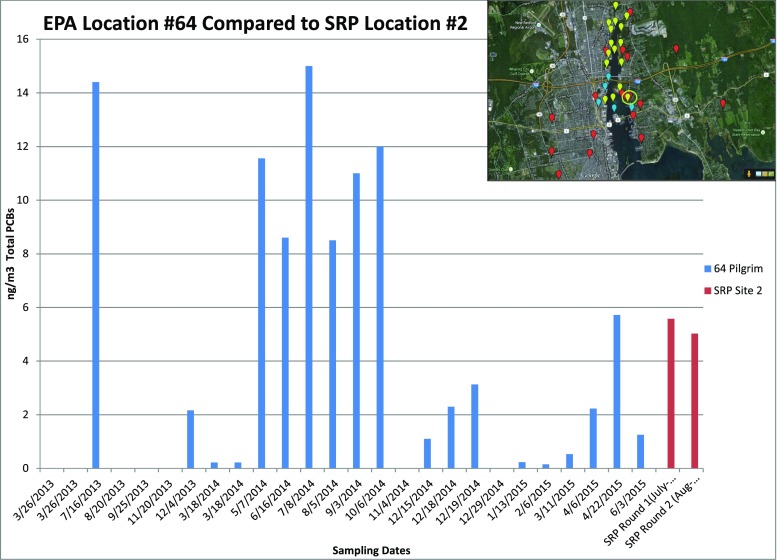
Comparison of historical EPA ambient air-monitoring PCB concentrations to concentrations measured at host sites (US EPA 2017)

**Fig. 7:**
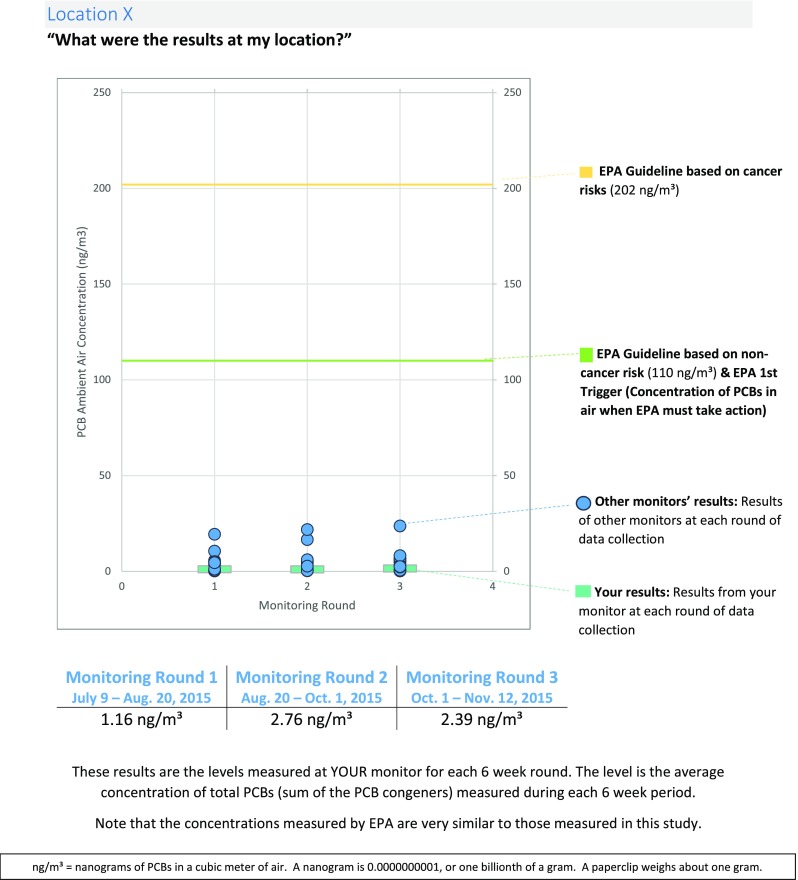
Example of Tailored Results Mailings to Monitor Hosts

Participants became increasingly vocal as they worked through the dataset, asking each other questions and sharing opinions about their observations. Two hosts who had never met before discussed the differences in their ambient air concentrations across sites. They posited that this could be attributed to prevailing winds and a nearby dewatering facility associated with the remedial dredging. Hosts began to identify trends in the data, commenting that some of the concentrations were slightly higher in the second round of monitoring than the first round. Without seeing a map of the locations, they accurately concluded that the Fairhaven monitors nearest to the harbor had higher concentrations than monitors on the New Bedford side. They also recognized that some sites had data below the level of detection.

As monitor hosts shifted from data discovery to understanding and discussion, Toxics Action community organizers brought the group back together to discuss reactions in a structured manner. As a group, hosts, Toxics Action, and BUSRP discussed and addressed the questions written on sticky notes by theme. Questions that could be immediately answered were addressed, such as defining PCBs, explaining a congener, and describing testing methodology. More technical questions concerning human health risks and causes for spatial differences in monitor concentrations were identified and tabled for the meeting 2 weeks later.

Anticipating questions about comparability of results to the US EPA monitoring data, BUSRP came prepared to show bar chart comparisons of monitoring sites from this study and the historical US EPA monitoring data from similar locations. Specifically, BUSRP prepared a map of the two compared locations in relation to the harbor and the historical US EPA data displayed chronologically, with the results of the two rounds of this monitoring project included, as shown in Fig. 6.

The concentrations measured in the first two rounds of this project were comparable with, and for the most part, slightly lower than the historic ambient air concentrations measured by the US EPA (US EPA 2017). Initially, members of BUSRP believed that the comparability of the results would be comforting to the monitor hosts. However, at this workshop, the comparison raised more questions than answers as to what the two different datasets measured, how they could be compared, and to what regulatory guidelines they could be compared. Monitor hosts asked questions about whether the US EPA data was representative of the “true” ambient air PCB concentrations and which data they should “believe.” Because of this discussion, comparisons between the two monitoring methodologies and relevant regulatory standards were highlighted as a topic for the next workshop.

Between workshops, BUSRP compiled questions raised by the participants and created a PowerPoint presentation to address those questions that could be answered. Many of the questions related to conclusions about the data in relation to human health risk and what could be done moving forward. The presentation included a brief overview of PCBs and ways that they can be categorized (i.e., congeners, Aroclors, homologs). The differences between regulations, standards, and guidelines were defined, and those that were applicable in NBH were identified and explained as compared to the measured concentrations. The second workshop lasted for 2 hours.

### Evaluations from first workshop

Seven of the ten attendees completed and returned the distributed written evaluations. All reported an ability to better understand numeric data on ambient air PCBs. They attributed their progress in this area to the discussion with the environmental health experts. They noted that the slow, clear, and logical explanations and sharing of the other hosts’ results were helpful. One participant noted that the raw data sheets looked like the US EPA data they had previously seen. When asked if participants gained experience understanding numeric data, units, and concentrations, four strongly agreed, and three agreed. As a follow up question, we asked if the workshop helped them gain skills to interpret numeric data, units, and concentrations. Three strongly agreed, and four agreed with one noting that they “need more time and experience.”

Participants were also asked whether they became more confident comparing the BUSRP and US EPA data on ambient air PCBs. Two participants strongly agreed, two agreed, and three reported that they neither agreed nor disagreed. One such participant elaborated, “[It was] not enough time for me to take this in as well as it needs to happen so I learn it.”

We asked participants to comment on what was the most interesting or valuable thing they would take from the session. Four commented on the utility of the skills learned in the session: “seeing the actual numbers from the sampling and what areas are seriously impacted;” “learning about the scientific processes of collecting the data and the effort to analyze, and the effort made to so I can understand it in a useful/helpful way;” and “knowing results in different areas and comparisons.” Another participant commented on the atmosphere as, “a much more trusting environment than any of the EPA meetings.” This was a sentiment expressed by several participants in the course of our workshops. Additionally, conversations revealed that participants did not realize the amount of work required by researchers to examine data for patterns over time or space. Seeing the raw data helped them generate their own questions and appreciate what it takes to answer such questions.

### Follow up: second workshop

There were seven attendees at the second workshop, all of whom had attended the first workshop. To begin, participants briefly recapped the previous meeting and shared what they took away from the experience. A range of emotions and thoughts were shared with some participants feeling empowered by the experience of the last workshop, and others sharing to the realization that studying the exposures and health concerns around the harbor was a much more complicated proposition than they had previously realized.

After having time to reflect on the previous workshop, monitor hosts were eager to work through the data interpretation as a group and to better understand how their data compared to data collected at other PCB-contaminated areas. Participants asked if there was any comparable data from the Hudson River Superfund site in New York facing PCB contamination and if their air concentrations were similar. They also asked if these data collection methods were considered “valid.” BUSRP members explained that Iowa SRP had a long history of using these passive samplers and the methods are well documented in the scientific literature (Martinez et al., 2017)(Herkert et al., 2016)(Ampleman et al., 2015).

BUSRP members proceeded with the slide presentation, introducing the recently received third round of data and responding to questions raised at the prior meeting. Participants did not have any questions in this workshop related to explaining the differences between congeners, Aroclors, and homologs, or sources of PCBs. One slide visually displayed the most abundant congeners measured through this project and stated that “none of these are well studied for health effects.” At this point, several participants asked. One asked, “So you have no data?” Another quickly followed up, “So it doesn’t mean they’re not toxic, it’s just they haven’t been studied?” The hosts’ level of concern elevated at this point. They had been hopeful that this study would give them definite, clear-cut answers about the relationship between the PCBs from the harbor and health risk. This was a turning point at which they began to realize that the scientific literature and expertise available may not able provide conclusive answers to all their questions.

As the presentation progressed into a section comparing federal concentration regulations, state air guidelines, project-specific limits, and trigger levels, participants became increasingly animated, asking many questions. Early questions involved the comparability of different monitoring methods, and different time scales: “24 hour average versus annual average? Do we divide our numbers by 365? How can you compare six-week sampling to these times?”; “What does a 24 hour average mean?”; “Our air monitors – would you not consider them as a snapshot? So on the readings, is it possible that one day it was high?”; “Do you have data on another area of Massachusetts- what their levels as a baseline would be? How much riskier is it here?”

Monitor hosts turned their frustration in this section towards regulatory agencies, suggesting that the different standards and guidelines were purposely complex to be exclusionary.

### Evaluations from second workshop

Five of the attendees completed and returned the written evaluations after the second workshop. When asked if they could better understand the role numeric data plays in regulations and guidelines on ambient air PCBs after this session, two responded “No,” and three responded “Yes.” Those who answered “Yes” commented that the “PowerPoint presentation graphs more easily identified levels” and that “verbal explanation always helps me. PowerPoint excellent... Questions and discussion also very helpful.”

Most of the participants reported gaining experience understanding congeners, homologs, and Aroclors. Two agreed that they gained skills to interpret regulations and guidelines on ambient PCBs and two neither agreed nor disagreed. One disagreed with this and disagreed that they became more confident in comparing this project’s data to the US EPA’s data on ambient PCBs. Three other participants agreed that they were more confident in this comparison and one neither agreed nor disagreed.

Participants were asked to comment on the most interesting or valuable thing they took from the session and how it would be useful to them. One noted that it would be useful to continue testing air in their area, one expressed surprise about how complex the topic was (a recurrent theme), and a third said that they gained a much better understanding of “how to dissect the air charts.”

### Report-back mailings

The first mailing contained data from the first three rounds of monitoring and was mailed in summer 2016. The second mailing included data from all four rounds and was mailed in fall 2016. The concentration from each location was displayed visually and numerically in the context of the location results, as displayed in Fig. 7. For comparison, the US EPA’s guideline levels for protection of human health were included. The second mailing also included a summary of a peer-reviewed article recently published by Iowa SRP and BUSRP researchers (Martinez et al., 2017). This paper identified NBH as the source of PCBs in the ambient air, and that the concentrations measured in this region were higher than those found at other PCB-contaminated sites.

### Host interviews

Monitor hosts were contacted via email and/or telephone. Of the 17 monitor hosts, we contacted seven, six of whom agreed to be interviewed. Of the six hosts, three had participated in both workshops and three had not participated in either workshop. The seventh host indicated that she was not comfortable participating in an interview. The eighth host died between the completion of the monitoring efforts and beginning of outreach for host interviews. Nine monitor hosts did not return our calls or emails.

When prompted to describe their memory of the results, four of the participants expressed relief and comfort that the monitors at their location indicated concentrations below the US EPA guideline levels. One participant reported that this did not assuage their concerns: “My comparison with others’ was lower, but that doesn’t necessarily mean that I’m happy that I have PCBs in the air near me.” Another stated that there was trouble with the air quality and that, “there’s definitely something drastic going on.” The hosts’ levels of concern regarding air concentrations did not correlate with individual results of PCB ambient air concentrations.

Of the 17 original monitor hosts (one individual hosted two monitors at different properties for a total of 18 locations), ten participated in the first workshop, seven in the second workshop, and six participated in the follow-up phone interviews, as shown in Table 1. Three of the hosts who were interviewed did not attend the data workshops and two said that it was due to scheduling conflicts. One did not attend because they did not believe they had anything “to add.”

**Table 1:** Number of participants in each stage of the project

Round/event	Round 1 monitoring	Rounds 2–4 monitoring	First workshop	Second workshop	Phone interviews
Number of participants	17	18	10	7	6

## Discussion

The objective of the two workshops was to provide the New Bedford Harbor (NBH) monitor hosts the time and resources to feel ownership of the data generated with their participation. This concept is very familiar in environmental health research where exposure assessment or human biomonitoring is conducted (Silent Spring Institute, 2017). It is widely recommended that individuals have the option of receiving their exposure results (Nelson et al., 2009). Prior knowledge of the setting and comments made at workshops and in evaluations highlight historical distrust of the US EPA and governmental agencies and a shared concern that hosts did not know what information to trust. Using the Statistics for Action (SFA) materials for initial data report-back provided a framework for discussing complex scientific concepts.

The evaluations from the first workshop demonstrated that the materials and framework provided by SFA for communicating science to communities was effective, but not sufficient. Participants reported greater confidence in their data analysis and that the environment created was trusting and inviting to collective learning. By allowing monitor hosts to see their raw data first, they were empowered to define their questions and draw their own conclusions rather than relying on the “expert” analysis. This approach allowed volunteer participants to have a more hands-on approach in the scientific process and to better grasp uncertainties inherent in science. The hosts compared the workshop to prior experiences with the US EPA and reported that this format inspired trust between the participants and organizers and in the conclusions hosts drew from the data.

To ensure that future uses of the First Look at Technical Documents are successful, we offer several recommendations, italicized here with explanations provided. *First*, *ensure that the meeting space is optimal for collaboration.* An ideal location would have large tables around which participants can gather and spread their materials. It should have good lighting and acoustics that allow the group members to clearly hear one another. The room in which these workshops were held was large with vaulted ceilings and poor lighting, resulting in challenging acoustics. Having a location in which participants can hear each other and see the data easily may further encourage discussion and minimize frustration.

*Adding another workshop shortly ahead of the data report-back meeting would also be useful to provide a contextual framework in which participants can discover their own data*. There were logistical challenges (e.g., host and facilitator availability, identifying suitable locations, tight timelines), which prevented the execution of this type of event. In this instance, the pre-workshop could have reviewed existing local ambient air data collected by the US EPA, as well as ambient air data from other Superfund sites and areas throughout the country for comparison. At our workshops, participants who had PCB concentrations higher than their fellow monitor hosts were highly concerned. Comparisons at the end of the workshop to the US EPA data showing large ambient air concentration reductions over the last decade and to health-based regulatory standards that were higher than their concentrations did little to mitigate their concerns. Providing them with these pieces of information shortly before they learn about their own ambient air concentrations may help lessen the shock felt by those who have higher concentrations relative to their peers. Although this information was presented at the end of the first workshop, the message seemed to have been overshadowed by fear that higher comparative concentrations meant higher direct risk to their health.

One of the more useful tools added to this workshop was a map with bar charts displaying the monitors’ ambient PCB concentrations. Several hosts who had never met one another sought one another out when they realized there was someone just a few blocks away from them with a monitor. They worked together to look through the data and to brainstorm explanations as to why one of their properties seemed to have consistently higher concentrations than the other. This visual tool was particularly useful to bring the monitor hosts together to discuss patterns that they had noticed within their own location over the three rounds as compared to all the monitors across the harbor.

The second workshop left the monitor hosts less confident in their data analysis skills and their ability to integrate their personal ambient air concentrations into the broader context of various regulatory guidelines. Hosts struggled to compare their data to these guidelines that involved different averaging times, collection methods, and applications. Hosts were presented with federal occupational air guidelines and regulations, state ambient air guidelines, and project-specific allowable ambient limits and risk-based goals so that they could compare their monitor’s results to relevant regulatory benchmarks. The differences in these guidelines, and the number of inputs that went into calculating each of them, were overwhelming to the monitor hosts, prompting many questions about how much math would be needed to make their data comparable and relevant to human health risk. These guidelines seemingly frustrated monitor hosts because they were unprepared to interpret the different methods of collection and averaging times.

The difference in the workshop format may have played a role in the hosts’ confidence. The second meeting was PowerPoint-driven, with attendees taking notes on printouts of the slides. The intention to answer hosts’ questions raised at the first workshop seemingly necessitated this format to provide the information in a streamlined and timely fashion. *In the future*, *it may be beneficial to create another workshop that allows for more independent discovery of these regulatory levels to continue the style of the first workshop.* This could involve a take-home worksheet after the first workshop that guides hosts through online research of regulatory standards and the data that go into them. The independent research may result in participants arriving better prepared to the second workshop. Scheduling only 2 weeks between workshops was not sufficient time for the BUSRP team to adequately prepare materials in response to the questions.

The surrounding neighborhoods of NBH are home to many residents that speak other languages. *If the workshops were offered to the broader population*, *simultaneous interpretation to allow for a bi- or multi-lingual workshop would have been necessary*. The fact that not all hosts participated in the workshops and interviews may be the result of the recruitment process for hosts or unwillingness to spend more time in meetings discussing PCBs. Community members identified people they knew in certain cross sections of the region who may be willing to allow a monitor to be hung on their property. Many of these hosts were willing to help, but were not interested in attending the community meetings, or being involved beyond hosting a monitor. Many residents involved in the project have spent years attempting to follow the NBH Superfund clean-up progress and are understandably frustrated at the lengthy process, a fact that also may have contributed to the participation rates. More lead-up time identifying community members who are concerned and/or interested in attending data report-back events may result in increased participation.

Finally, it is very possible that residents surrounding NBH experience PCB-related fatigue. The long-term remediation process has created divides within the local community in terms of their trust of regulatory agencies, other community groups, and academics. These groups within the community have different needs and desires regarding the information they would like and the action they would like to take place to address the harbor contamination. A combination of differing needs and burn-out related to the harbor contamination may also have played a role in hosts’ interest in attending and engaging in the data report-back workshops.

### Limitations

There were several limitations in this study. First was a small sample size and potential selection bias of volunteer monitor hosts. Volunteers who hosted the air monitoring equipment were either members of HARC (most of whom attended the initial meeting in Fairhaven), or individuals who were identified by HARC members. Those who attended the first meeting in Fairhaven were primarily the hosts who continued to attend the workshops and participate in the follow-up phone calls. A more concerted effort to identify volunteer hosts who will be invested in the results of the air monitoring may lead to higher participation rates in future projects. Volunteers who did actively participate in the workshops and follow-up phone calls may have been more likely to have a preexisting interest in local exposure to PCBs and to have some history of researching the science and/or policy related to the NBH cleanup efforts.

## Conclusions

The structure of the First Look at Technical Documents workshop was effective for data discovery by the New Bedford Harbor (NBH) monitor hosts and was well received by the attendees. Most participants reported increased confidence in their ability to understand sophisticated environmental PCB data and to compare different data sets. The environment also fostered a trusting atmosphere for communal discussion, and to build community relationships.

An important outcome of the project was validation for Hands Across the River Coalition (HARC) and certain hosts that the NBH contamination contributes to concentrations of airborne PCBs, a long-held belief by many residents of the surrounding neighborhoods. The air monitor results demonstrate that NBH is the single largest continuous source of airborne PCBs ever measured from natural waters in North America (Martinez et al., 2017). Some hosts did not know one another before the project and worked together during the workshops to analyze the results and discuss next steps. The project supported community-building opportunities and clarified the need for future research to evaluate the human health risk associated with the airborne PCB concentrations.

BUSRP Community Engagement partners, Toxics Action, Alternative for Communities and Environment, and Iowa SRP gained valuable experience in implementing Statistics for Action materials in the context of human exposure data report-back to a community. Overall, the workshops were successful in communicating complex exposure data, and the materials were well suited for community members with varying levels of experience with science and PCB research.
